# Effect of intentional daily liter of plain water on total fluid and caloric intake among free-living adults

**DOI:** 10.1371/journal.pone.0352203

**Published:** 2026-07-21

**Authors:** Xiujing Zhao, Whitley C. Atkins, Brendon P. McDermott

**Affiliations:** 1 Heat & Hydration Optimization (H2O) Lab, Fayetteville, Arkansas, United States of America; 2 Exercise Science Research Center, Fayetteville, Arkansas, United States of America; 3 Department of Health, Human Performance & Recreation, University of Arkansas, Fayetteville, Arkansas, United States of America; 4 Department of Health, Exercise & Sports Sciences, University of New Mexico, Albuquerque, New Mexico, United States of America; United States Olympic & Paralympic Committee, UNITED STATES OF AMERICA

## Abstract

While previous studies have examined short-term water consumption and corresponding energy intake, few have investigated the effects of a longitudinal intervention in non-obese, young adults. We aimed to evaluate the effect of a 6-week daily liter of plain water consumption on fluid and caloric intake among free-living healthy adults. Thirty-eight young healthy adults (25 ± 6 y, 1.67 ± 0.09 m, 73.0 ± 16.7 kg, 25.8 ± 4.2 of body mass index) were provided two 500mL bottles of water per day and asked to consume them for six weeks. They were provided no further direction on fluid consumption during the study. Participants recorded 24-hour total food and fluids consumed and collected urine across three time points of baseline, 3-week (mid), and 6-week (post). Twenty-four-hour total fluid, plain water, sugar-sweetened beverages, caloric intake, urine specific gravity (USG), urine osmolality (U_osm_), and urine volume were measured at all three time points. Hydration status was assessed via 24-hour USG and U_osm_. Twenty-four-hour total fluid intake was significantly increased at mid (p = .040) but not post (p = .426). Twenty-four-hour total plain water intake was significantly increased at mid (p = .028) and post (p = .049) time points. No significant differences were observed in 24-hour total sugar-sweetened beverage intake across time points (p = .210). Twenty-four-hour total caloric intake was significant reduced at the post (p = .048) but not at mid (p = .257). Twenty-four-hour total caloric intake was not associated with 24-hour total fluid intake (r = .052, p = .618), total plain water intake (r = −.049, p = .636), and total sugar-sweetened beverages intake (r = .143, p = .170). No significant differences were found in 24-hour USG (p = .075) and total urine volume (p = .491) among time points, but U_osm_ significantly decreased at mid (p = .001). In conclusion, a moderate increase in plain water intake may prompt a decrease in daily caloric intake and thus facilitate weight management.

## Introduction

Water is essential for maintaining physiological homeostasis and supporting optimal function [1–3]. Although the human body is composed of approximately 45–75% water [4], fluid deficits equivalent to 1–2% of body weight can adversely impact health [5], cognitive function [6], and exercise performance [7,8]. Severe dehydration, such as a 20% reduction in total body water loss, can be life-threatening [9]. Given that body water loss occurs inevitably through the kidney, lung, gastrointestinal tract, and skin, regular water replacement is necessary to compensate [10]. Thus, adequate daily water intake is vital for sustaining health.

Previous literature demonstrates that meeting daily adequate fluid intake guidelines is associated with positive health outcomes [11–16]. Leading public health organizations, such as the National Academy of Medicine (NAM) and the European Food Safety Agency (EFSA), have disseminated adequate fluid intake guidelines based on epidemiological data from national food surveys [17,18]. In the United States, the NAM recommends an adequate daily total fluid intake of 3.7 and 2.7 litres (L) for male and female adults (e.g., 20% of daily fluid consumption contained in food), respectively [17]. The NAM fluid intake guidelines were established based on the median total fluid intake from a national survey in the U.S. [17]. At the same time, the EFSA guidelines were derived from observed fluid intake and relative to a “desirable” 24-hour urine osmolality of < 500 mOsm/L, which is based on the renal solute load from national dietary surveys across multiple European countries. EFSA advises a total daily fluid intake of 2.5 and 2.0 L for male and female adults, respectively [18]. Although individual daily water needs vary due to the innate nature of fluid requirements and individualization (e.g., age, sex, physical activity level, health status, etc.) across varied environments, following the adequate daily fluid intake guideline is beneficial to maintain optimal hydration and normal physiological function [2,19].

Beyond its basic functions, increases in water intake may contribute to reducing energy intake and, therefore, may promote weight management [20]. Plain water consumption is associated with reduced energy intake due to the replacement of high-calorie beverages (e.g., sugar-sweetened beverages) [20]. High-calorie beverage consumption results in excessive energy intake, which significantly increases body weight and the risk of obesity [21]. It is important to note that individuals who consume high-calorie beverages do not reduce food intake to compensate for the extra calories [22,23]. Studies have shown that replacing high-calorie beverages with plain water can be an effective way to reduce energy intake by approximately 200–235 kcal [22,24–26]. Individuals who consume food with plain water rather than high-calorie beverages consistently report lower total energy intake [22,26]. Additionally, water consumption can enhance short-term satiety and reduce hunger sensation [27,28], further supporting its role in weight management and obesity prevention.

It is well-established that water consumption is a direct and effective strategy for maintaining fluid balance and physiological function [29]. While previous studies have examined short-term water consumption and corresponding energy intake, most investigations have been cross-sectional or acute in nature and have primarily focused on overweight or obese populations [22,28]. As a result, the extent to which a sustained increase in plain water intake influences fluid consumption behaviors and caloric intake in generally healthy, free-living adults remains unclear. Understanding these relationships in a healthy population is important for identifying early, modifiable behaviors that may support long-term health and weight management. Therefore, the purpose of the present study was to examine the effect of a 6-week prescribed increase in daily plain water intake on fluid and caloric intake among free-living, healthy adults. We hypothesized that increasing plain water intake would lead to an increase in total fluid consumption and a reduction in daily caloric intake.

## Materials and methods

### Description of participants

The present study employed a longitudinal intervention design to examine the effects of intentionally consuming 1 liter of plain water daily for six weeks on participants’ fluid and caloric intake. The study was conducted in a free-living environment between October 2022 and November 2023, environmental conditions were not controlled, and all assessments were performed at the Heat & Hydration Optimization Lab. Thirty-eight young, healthy adults (female: 29, male: 9; age range: 18-47y) were involved in our study. Our convenience sample participant demographics are included in Table 1. Before recruiting participants, this study was approved by the University of Arkansas Institutional Review Board (IRB#: 2102314312). Participants were recruited via campus e-mail, verbal announcements at group meetings, and class advertisements. Participants were eligible if they were over 18 years of age, had no diagnosed chronic medical conditions (e.g., cardiovascular, metabolic, or renal diseases), and were not taking prescription medications that alter kidney function or fluid balance. Minor acute conditions (e.g., common colds) did not preclude participation. All participants self-identified as generally healthy. All recruited participants met the inclusion but not exclusion criteria, and no participants were excluded after screening. All participants reported to our laboratory and provided written informed consent. Prior to signing the informed consent form, participants were instructed to read it thoroughly and encouraged to ask clarification questions about the purpose, risks, benefits, time commitment, and procedures of participation. Participants were free to withdraw from the study at any time without consequence. Data collection occurred between October 2022 and November 2023. Participants were free-living with specific direction for them to continue daily habits.

**Table 1:** Sample characteristics in data collection (n = 38).

Characteristics	mean ± SD [min, max]
Age (y)	25 **±** 6 [18,47]
Height (m)	1.67 **±** 0.09 [1.50, 1.83]
Mass (kg)	73.0 **±** 16.7 [45.0, 116.6]
Body mass index (kg/m^2^)	25.8 **±** 4.2 [20.0, 39.2]

### Procedures and intervention

Participants first completed baseline assessments, including 24-hour urine collection and dietary/fluid intake recording, prior to the initiation of the intervention. Following baseline data collection, participants were instructed to consume an additional 1 L/day of plain water for six weeks, on top of their habitual fluid intake. The prescribed volume was standardized as two 500 mL servings to be consumed at consistent times throughout the day (e.g., morning and afternoon), although participants were allowed flexibility to accommodate their daily routines. Participants were instructed to maintain their usual dietary habits, physical activity levels, and beverage consumption patterns outside of the prescribed water intake. No specific restrictions were placed on other fluid or food intake. Compliance was encouraged through verbal instructions and periodic reminders, but fluid intake was not directly supervised given the free-living nature of the study.

Participants were informed that the study examined fluid intake and hydration-related behaviors as part of the informed consent process. While participants were instructed to maintain their usual dietary and fluid intake habits outside of the prescribed intervention, their awareness of the study purpose may have influenced their fluid consumption.

### Assessment of 24-hour fluid, plain water, sugar-sweetened beverages, and caloric intake

The present study utilized food and fluid consumption logs to record participants’ 24-hour food and fluid consumption at three time points (baseline, 3-week [mid], and 6-week [post]) during our six-week intervention. Food and fluid consumption logs have been used to accurately collect information on food and fluid consumption [30–33]. During the preliminary lab visit, researchers educated participants on data recording responsibilities and answered questions prior to participants leaving the laboratory. For a 24-hour food log entry, participants recorded the meal type (e.g., breakfast, lunch, dinner, or snack), food type, amount, and cooking method, and time of day. Regarding the 24-hour fluid log entry, participants recorded the fluid type and volume during different time points (e.g., after lunch, dinner, before sleep, after waking up, breakfast, after breakfast, and lunch). Fluids were selected from a list and were grouped into eight classes: water; coffee/tea/hot chocolate; milk/soy milk/drinkable yogurt; 100% fruit juice/fruit drink/lemonade/other sweet drink (e.g., smoothie); fizzy drink (e.g., Coca Cola, Fanta, etc.); energy drink (e.g., Red Bull, Rockstar, etc.); sports drink (e.g., Gatorade, Powerade, etc.); protein drink/protein shake (e.g., muscle milk, Bio-Engineered Supplements and Nutrition, etc.). Total fluid and caloric intake were quantified using nutrition software (Nutritionist Pro version 7.8.0, Axxya Systems, Redmond, WA, USA). Total fluid intake included total fluid consumed and dry food water content. Total plain water intake was calculated based on the 24-hour fluid log. Total sugar-sweetened beverage intake was quantified from the same 24-hour fluid log, following the definition of sugar-sweetened beverages provided by United States Dietary Guidelines [34].

### Assessment for hydration status by urine biomarkers

In line with data collection on 24-hour food and fluid consumption, 24-hour urine was collected at three time points (baseline, mid, and post). Participants were provided with two urine collection containers to facilitate complete 24-hour urine collection and to ensure adequate storage capacity throughout the collection period. Participants were instructed to collect all urine voids across the 24-hour period in these containers. Researchers assessed and recorded 24-hour urine specific gravity (USG), urine osmolality (U_osm_), and total urine volume immediately after the urine jug was returned. The tare weight of each urine collection container was recorded prior to distribution, and this value was subtracted from the total measured weight following collection to determine net urine volume. During each assessment for urine biomarkers, 24-hour USG was determined using a refractometer (Master-SUR, Atago Co., Ltd., Tokyo, Japan). Twenty-four-hour Uosm was measured via freezing point depression (3250, Advanced Instruments Inc., Norwood, MA, USA).

Hydration status was assessed via 24-hour USG and U_osm_. The threshold between euhydration and hypohydration was set as 1.020 [35] and 720 mOsm/L [36] for 24-hour USG and Uosm, respectively.

### Statistical analysis

Data were analyzed with GraphPad Prism software (Version 10.2.3) and R (Version 4.5.3) in RStudio. Descriptive characteristics were calculated for all study variables and reported as mean ± standard deviation (SD). An a priori power analysis was conducted using G*Power (version 3.1.9.7) to determine the required sample size for the repeated-measures ANOVA (N ≥ 34) and correlation (N ≥ 38) analyses. Normality of all variables was assessed using the Shapiro–Wilk test and visual inspection of Q–Q plots and histograms; no significant departures from normality were observed (all p > 0.05). To examine changes over time (baseline, mid, and post), linear mixed-effects models were employed to account for within-subject variability across repeated measures, with participant included as a random effect. When a significant main effect of time was identified, post hoc pairwise comparisons were conducted using Tukey’s adjustment for multiple comparisons. To assess relationships between variables measured across repeated time points, repeated-measures correlation analyses were performed to examine the within-individual association between 24-hour total caloric intake and total fluid intake, total plain water intake, and total sugar-sweetened beverage intake. Actual p-values are reported where possible. Statistical significance was set α priori at p ≤ 0.05.

## Results

### Sample characteristics and intervention description

Table 1 provides the participant characteristics. According to the NAM guidelines for daily total fluid intake, the percentage of participants who met the guidelines was 3.1%, 11.8%, and 18.8% at baseline, mid, and post, respectively.

### 24-hour fluid, plain water, sugar-sweetened beverages, and caloric intake

**Fig 1:**
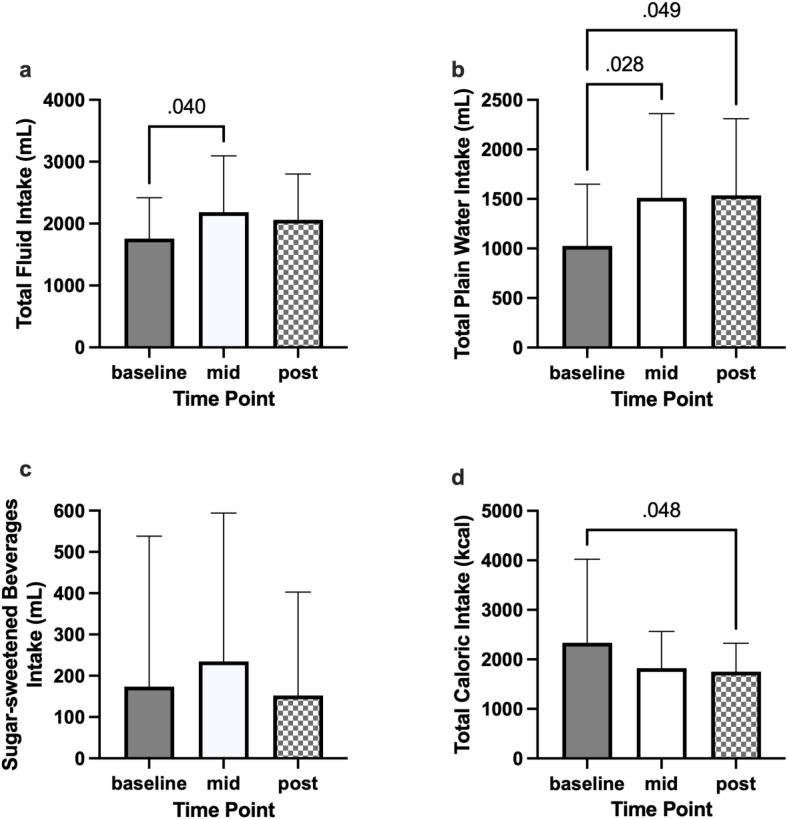
Changes in 24-hour total fluid, total plain water, and total caloric intake across baseline, mid, and post. a): 24-hour total fluid intake; b): 24-hour total plain water intake; c): 24-hour sugar-sweetened beverage intake d): 24-hour total caloric intake. Data are present as mean and SD.

There was a significant effect of the daily liter of plain water consumed on 24-hour total plain water intake (p = .027, Fig 1a). A post-hoc analysis showed that 24-hour total fluid intake was significantly increased at mid (2185 ± 910 mL) compared to baseline (1760 ± 659 mL, p = .040), whereas no significant differences were observed between baseline and post (p = .426). In addition, there was a significant increase in daily water on 24-hour plain water intake (p = .003, Fig 1b). Daily total plain water was significantly increased at mid (1512 ± 850 mL, p = .028) and post (1537 ± 773 mL, p = .049) compared to baseline (1027 ± 623 mL). No significant differences were observed in 24-hour total sugar-sweetened beverage intake across the three time points (p = .210, Fig 1c). Twenty-four-hour total caloric intake was significantly reduced at post (1751 ± 577 kcal, p = .048) but not at mid (1824 ± 742 kcal, p = .257) compared with baseline (2335 ± 1686 kcal, Fig 1d).

Additionally, no significant correlations were observed between 24-hour total caloric intake and 24-hour total fluid intake (r = –.027, p = .835), total plain water intake (r = –.098, p = .456), or total sugar-sweetened beverage intake (r = –.025, p = .849).

### Hydration biomarkers

**Fig 2:**
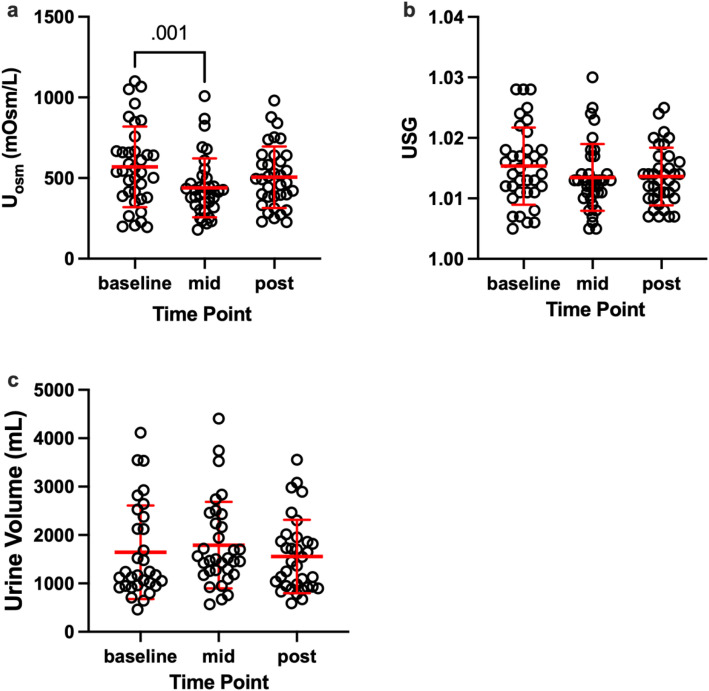
Changes in hydration status based on urine biomarkers across baseline, mid, and post. a): 24-hour urine osmolality (U_osm_); b): 24-hour urine specific gravity (USG); c): 24-hour total urine volume. Data are shown as means ± standard deviation (red cross) with individual data.

A daily liter of water consumed had a significant effect on 24-hour U_osm_ (p = .003). U_osm_ was significantly decreased at mid (439 ± 183 mOsm) compared to baseline (569 ± 250 mOsm, p = .001, Fig 2a), whereas no significant differences were observed between baseline and post (505 ± 191, p = .222, Fig 2a). In contrast, no significant differences were found in 24-hour USG (p = .075, Fig 2b) and 24-hour total urine volume (p = .491, Fig 2c) among time points.

Hydration status was determined using the threshold of USG (1.020) [35] and Uosm (720 mOsm/L) [36]. Based on average urine biomarkers, our participants were euhydrated throughout our study: baseline (USG: 1.015 ± 0.006, U_osm_: 569 ± 250 mOsm/L), mid (USG:1.013 ± 0.006, U_osm_: 439 ± 183 mOsm/L), and post (USG: 1.014 ± 0.005, U_osm_: 505 ± 191 mOsm/L).

## Discussion

Our study investigated the effect of a six-week intervention involving intentional daily consumption of 1 liter of plain water on fluid intake, caloric intake, and hydration status among free-living, healthy adults, which revealed several important findings. First, a six-week daily liter of plain water consumption significantly increased 24-hour total fluid intake, particularly total plain water intake, without altering total sugar-sweetened beverage intake. Second, over the course of our study, participants significantly reduced 24-hour total caloric intake. Third, no significant differences were observed in hydration status, as assessed by urine biomarkers (24-hour USG, 24-hour total urine volume), although U_osm_ decreased at mid, but not post.

### 24-hour fluid, plain water, sugar-sweetened beverages

The six-week intervention, involving a daily intentional liter of plain water consumption, had a positive effect on 24-hour total fluid intake. Specifically, 24-hour total fluid intake significantly increased at mid compared to baseline. However, this increase returned to baseline level by post. This pattern suggests that the intervention initially increased total fluid intake; however, this effect was not sustained at post. While participants were instructed to consume an additional1 L/day of water, the reduction in total fluid intake from midpoint to post-intervention may reflect a compensatory decrease in fluid intake from other sources, variability in adherence to the prescribed water intake, or natural day-to-day variation in fluid consumption. Habitual fluid intake patterns were not directly assessed beyond baseline measures; therefore, changes over time cannot be definitively attributed to a return to habitual intake. Therefore, the intervention appears to have had a transient effect on total fluid intake rather than a sustained increase. In contrast, plain water intake remained significantly elevated at both mid and post, indicating a more sustained behavioral change in water consumption despite the attenuation in total fluid intake. However, the modest increase of 238 mL in 24-hour total fluid intake was not sustained at post-intervention and is unlikely to confer meaningful physiological or clinical benefits, particularly given that participants were already euhydrated at baseline. Thus, the primary impact of the intervention appears to be a behavioral shift toward increased plain water consumption rather than an improvement in hydration status. Although the proportion of participants meeting National Academy of Medicine (NAM) total fluid intake guidelines increased from 3.1% at baseline to 18.8% at post, the majority remained below these recommended intake levels. Notably, participants were already euhydrated based on urinary biomarkers, indicating that meeting NAM intake targets was not required to maintain adequate hydration in this sample. This highlights that while the intervention improved adherence to these intake recommendations, the magnitude of change was modest at the group level. The NAM guidelines are intended as population-level recommendations, and their interpretation should be considered alongside individual hydration status, which in this study indicated that participants were already generally euhydrated. Thus, NAM guidelines should be interpreted alongside physiological hydration markers when evaluating hydration adequacy at the individual level.

The increases in 24-hour total plain water intake were more pronounced than those in total fluid intake. Twenty-four-hour total plain water intake significantly increased at both mid and post compared with baseline. In contrast, our data showed no significant changes in 24-hour total sugar-sweetened beverage intake across time points. Since previous research has demonstrated that higher water consumption was associated with reduced caloric intake [19,24,25], the 510 mL increase in 24-hour total plain water intake observed in the present study may, at the group level, have contributed to the reduction in total caloric intake. However, no significant individual-level association was detected between these variables. Previous evidence indicates that consuming 500 mL of plain water before meals, alongside a hypocaloric diet, significantly reduces caloric intake and results in a loss of 2 kg more in body mass than those who only followed the hypocaloric diet over 12 weeks [37]. Likewise, pre-meal water consumption has been shown to reduce caloric intake and can facilitate body weight loss, particularly in older adults [38,39]. Collectively, these findings support water consumption as a practical strategy for body weight maintenance or as an adjunct to facilitating weight loss.

### 24-hour total caloric intake

Twenty-four-hour total caloric intake was significantly reduced at post compared with baseline. In the current study, a self-reported 584 kcal reduction in 24-hour daily caloric intake occurred alongside a 510 mL increase in 24-hour total plain water intake at the group level. This finding aligns with prior evidence that greater water consumption is linked to reduced energy intake [19]. Two mechanisms may explain this reduction: displacement of sugar-sweetened beverages and a short-term enhancement of satiety. Previous research has shown that substituting plain water for sugar-sweetened beverages can reduce energy intake by approximately 200–235 kcal/day [24,25]. In the present study, although there was a minor 21.4 mL reduction in 24-hour sugar-sweetened beverage intake at post, this change alone could not account for the observed 584 kcal reduction in 24-hour caloric intake. This finding aligns with previous research that a higher proportion of plain water consumption is associated with reduced total daily energy intake and a smaller decrease in calories from total sugar-sweetened beverages [40]. Therefore, increased satiety may have played a primary role in reducing energy intake in the current study. Consistent with this notion, previous studies have suggested that increased water consumption may enhance satiety and reduce subsequent energy intake [27,28,41]. However, in the present study, the lack of a sustained increase in total fluid intake at post suggests that these mechanisms may not have been consistently maintained over the duration of the intervention, particularly in a population that was already adequately hydrated at baseline.

It is important to note, however, that alternative factors could have contributed to the observed reduction in caloric intake. These include seasonal variability, participant self-monitoring effects, or novelty associated with study participation, which may have influenced eating behavior independently of increased water consumption. Although total energy intake decreased at post, we did not analyze changes in carbohydrate, fat, or protein intake. Future studies could explore macronutrient-specific effects to determine which components of the diet are most affected by increased water consumption, and whether these effects differ by BMI or weight status. Therefore, the current findings should be interpreted as observational associations rather than causal effects.

The observed 584 kcal reduction in 24-hour total caloric intake is also clinically meaningful, corresponding to a potential weight loss of ~0.5 kg per week [42]. This finding is consistent with current obesity guidelines, which recommend daily energy reductions of 500–750 calories to achieve a safe and effective weight loss [43–46]. Our findings align with these guidelines, indicating water consumption may help reduce total caloric intake through a short-term enhancement of satiety. Thus, the present study supports the potential role of daily water consumption as a simple and practical dietary strategy for promoting healthier fluid intake patterns and reducing caloric intake.

### The relationship between total caloric intake and fluid intake

Investigating the relationship between total caloric intake and fluid intake is essential for understanding the potential role of water consumption in reducing daily total caloric intake and promoting weight loss. Although our intervention involving an intentional daily liter of plain water consumption significantly increased both total fluid and plain water intake while reducing total caloric intake, no significant correlations were observed between 24-hour total caloric intake and 24-hour total fluid intake or total plain water intake. In addition, 24-hour total sugar-sweetened beverage intake did not change across our study and was not associated with changes in 24-hour total caloric intake. This finding contrasts with previous research demonstrating that higher total sugar-sweetened beverage consumption is strongly linked to greater total energy intake [47]. Given that the limited research examining the relationship the relationship between total caloric and fluid intake (e.g., plain water and sugar-sweetened beverages), further studies involving larger sample sizes are warranted to clarify these associations.

### Hydration-related urine biomarkers

Water consumption is widely recognized as a practical approach for promoting optimal hydration status [1]. Previous studies report a strong association between fluid/water consumption and urinary hydration biomarkers, including 24-hour USG, Uosm, and total urine volume [10]. In the present study, 24-hour USG and total urine volume did not significantly change among the three time points, whereas Uosm significantly decreased at mid but not at post, mirroring the transient increase in 24-hour total fluid intake. Although hydration status did not change by conventional clinical thresholds, the lack of statistically significant changes in USG and U_osm_ may reflect the inherent variability of these biomarkers and their susceptibility to external influencing factors (e.g., recent fluid intake, dietary composition, and timing of urine collection), rather than a true absence of physiological response to the intervention. Previous studies have demonstrated that urinary hydration markers can exhibit considerable day-to-day variability and may be less sensitive for detecting subtle changes in hydration status in free-living, healthy populations [10,36,48].

Recently published literature strongly recommends using at least two biomarkers for a reliable assessment of hydration status [49]. Consistent with this recommendation, our study employed urine biomarkers, including 24-hour USG and U_osm_. The results suggest that participants were classified as euhydrated across all three time points [35,36], despite the fact that most participants did not meet the NAM guidelines for daily total fluid intake at baseline (96.9%), mid (88.2%), and post (81.2%) time points. Although participants were generally euhydrated at baseline, the intervention increased total fluid intake and improved adherence to NAM guidelines. These findings suggest that the primary utility of the intervention lies in improving adherence to recommended fluid intake levels and increasing plain water consumption, rather than altering hydration status in individuals who are already euhydrated.

### Limitations and future study

Results from the present study should be taken with caution based on several limitations. First, in this study, a free-living lifestyle design limited control over confounding factors such as physical activity and environmental conditions, which may have impacted fluid intake behavior. Participants were asked to maintain their regular exercise habits throughout the study; however, physical activity was not objectively measured or recorded, and therefore we cannot confirm that exercise behavior remained unchanged during the intervention. This lack of control or recording may have under- or overestimated our findings. Second, participants were aware that the study focused on hydration and fluid intake behaviors, which may have influenced their habitual drinking patterns independent of the intervention. This potential reactivity (e.g., behavioral modification due to awareness of being observed) may have contributed to changes in fluid intake, particularly during the early phase of the intervention. Third, the absence of a parallel control group may restrict causal inference, as changes observed over time cannot be attributed exclusively to the intervention. Fourth, the findings in this study were based on a small sample size, which may prevent broad extrapolation. Additionally, the relatively homogeneous hydration status of participants (predominantly euhydrated at baseline) limited our ability to perform subgroup analyses based on baseline hydration status; future studies including a wider range of hydration levels may help determine whether individuals with lower baseline hydration derive greater benefit from increased water intake. Additionally, the current analyses focused on group-level effects and did not formally examine individual differences in response; future research could explore responder and low-responder patterns to better understand variability in behavioral responses to water consumption. Fifth, 24-hour fluid and diet logs were self-report data, where methodological limitations may increase the potential risk of bias in reliability and validity of our findings, although the food and fluid logs have been validated [30–33]. Sixth, body mass was not measured at mid- or post-intervention; therefore, we cannot assess potential changes in body weight over the six-week period. Measuring body mass in future studies could provide additional insight into energy balance and help strengthen interpretation of hydration status alongside urine biomarkers. Finally, the sample was predominantly young and healthy adults, with a higher proportion of females (29 out of 38), which does not reflect broader population demographics. Sex differences in hydration behavior and thirst regulation, as well as age-related and health-related differences, may limit external validity. Therefore, future studies should employ larger and more representative samples, including children, older adults, and individuals with obesity and diabetes, to better evaluate how longitudinal increases in intentional water consumption influence daily fluid and caloric intake and related metabolic outcomes, consistent with broader considerations outlined in recent reviews of hydration behavior [50].

## Conclusions

The present study demonstrated that a six-week intervention involving intentional daily consumption of 1 liter of plain water significantly increased plain water intake, produced a transient increase in total fluid intake, and reduced total caloric intake among free-living healthy adults, while not significantly altering hydration status as assessed by urinary biomarkers. These findings suggest that simple, behavior-based water consumption strategies can effectively improve dietary intake patterns without requiring structured dietary restrictions. From a practical perspective, encouraging daily water consumption may serve as an accessible, low-cost intervention to support modest reductions in energy intake and promote healthier hydration habits in real-world settings. Given the observed variability in total fluid intake and the stability of hydration biomarkers, future translational efforts should focus on reinforcing sustained water consumption behaviors and identifying strategies to maintain long-term adherence in diverse populations.
