# Supplementary material for: Effect of intentional daily liter of plain water on total fluid and caloric intake among free-living adults
Source: PLoS One. 2026 Jul 21;21(7):e0352203. doi: 10.1371/journal.pone.0352203 (PMC13387533; doi:10.1371/journal.pone.0352203)
Supplement: Attachment — Submitted filename: Responses to reviewerss comments_PLOS One_R1.docx [file pone.0352203.s002.docx]

Dear Editor,

We sincerely thank you and the reviewers for the careful evaluation of our manuscript entitled “Effect of Intentional Daily Liter of Plain Water on Fluid and Caloric Intake among Free-living Adults” (Manuscript ID: PONE-D-26-07092). We appreciate the constructive comments and suggestions, which have greatly helped us to improve the quality, clarity, and rigor of the manuscript.

We have carefully addressed all reviewer comments and believe that the revised manuscript is substantially improved as a result. We greatly appreciate the time and effort invested by the reviewers and the editorial team in evaluating our work. We hope that the revised manuscript is now suitable for publication in PLOS One.

Thank you for your consideration.

Sincerely,

Brendon P. McDermott, PhD, ATC, FACSM

Department of Health, Human Performance and Recreation,

University of Arkansas

Email: [brendonm@uark.edu](mailto:brendonm@uark.edu)

Below, we provide a detailed, point-by-point response to each reviewer’s comment. All changes have been highlighted in the manuscript in accordance with the journal’s guidelines. Line numbers refer to the revised manuscript unless otherwise noted.

____________________________________________________________________________________

Review Comments to the Author

Please use the space provided to explain your answers to the questions above. You may also include additional comments for the author, including concerns about dual publication, research ethics, or publication ethics. (Please upload your review as an attachment if it exceeds 20,000 characters)

Reviewer #1: Introduction

What is the rational to study this population “less is known about the effects of a longitudinal intervention in non-obese, young adults.”?

We thank the reviewer for this important comment. We have strengthened the rationale by emphasizing the limited longitudinal evidence in healthy, non-obese populations and the importance of preventive behavioral strategies (Lines 75-82).

Method

“All participants self-identified as generally healthy.” is too broad.

We thank the reviewer for this comment and agree that the phrasing was overly broad. We have revised the description to clarify that participant health status was determined based on predefined inclusion criteria, including absence of diagnosed chronic medical conditions and use of medications known to affect fluid balance (Lines 98-101). The phrase “self-identified as generally healthy” has been removed to avoid ambiguity.

“1 liter of plain water daily for six weeks” means additional 1L to what they normally do? More information is needed in general. What was the instruction? Methods and procedures might not be enough.

We thank the reviewer for this important comment and agree that additional clarification of the intervention protocol is needed. We have revised the Methods section to explicitly state that participants were instructed to consume 1 L/day of plain water in addition to their habitual fluid intake. We also expanded the description of the instructions provided to participants, including how the water intake was distributed and how participants were asked to maintain their usual dietary and fluid consumption behaviors outside of the prescribed intervention (Lines 113-124).

Did participants know this was hydration study? If so, that typically impacts fluid intake as participants tend to increase water intake by knowing this was assessing hydration.

We thank the reviewer for this important observation. Participants were informed that the study involved fluid intake and hydration-related behaviors as part of the consent process. We acknowledge that this awareness may have influenced participants’ habitual fluid intake (e.g., increasing water consumption independent of the prescribed intervention). To address this, we have clarified this point in the Methods section (Lines 125-128) and acknowledged it as a potential source of bias in the Discussion (Lines 353-357).

“Pearson correlation was used to investigate the relationship between 24-hour total caloric intake and total fluid intake, total plain water intake, and total sugar-sweetened beverages intake.” Since multiple time points are included from one participant, repeated correlation is better.

We agree with the reviewer that a Pearson correlation does not account for the non-independence of repeated measures data. Accordingly, we have re-analyzed the relationships between 24-hour caloric intake and fluid variables using repeated measures correlation. The Results section have been updated to reflect these more robust statistical findings (Lines 178-182).

Discussion

“This pattern indicates the intervention may have temporarily promoted greater fluid intake, which diminished over time as participants returned toward their habitual fluid intake pattern.” means participants decreased the amount of water intake outside of additional 1 L from mid to post. Does this indicate the intervention was not successful? Please clarify!

We thank the reviewer for this insightful comment. We agree that the original wording may have implied ambiguity regarding the effectiveness of the intervention. We have revised the Discussion to clarify that the intervention was partially successful (Lines 230-238), specifically, it increased fluid intake during the early phase of the intervention, but this effect was not fully sustained over time. We further clarify that the attenuation in total fluid intake likely reflects behavioral adaptation or reduced adherence rather than complete intervention failure.

“Consistent with this notion, previous studies have demonstrated that water consumption enhances satiety and decreases subsequent energy intake, thereby contributing to weight loss [27,28, 41].” needs to be carefully interpreted as fluid intake was not increased at post.

We thank the reviewer for this important observation and agree that the interpretation of prior literature should be better aligned with our findings. We have revised the Discussion to avoid overgeneralization and to clarify that the proposed mechanisms linking water intake to reduced energy intake were not consistently supported in our study, particularly at the post-intervention time point (Lines 285-290).

Reviewer #2: Specific Comments (Suggested edits appear in CAPS)

Title: Should read “…TOTAL Fluid and Caloric Intake…”

Revised as suggested.

Abstract

To clarify the contrast, the first sentence should read (or similar) “While previous studies have examined SHORT-TERM water consumption and CORRESPONDING energy intake…”

We thank the reviewer for this helpful suggestion. We have revised the opening sentence of the Abstract to better emphasize the contrast with prior literature by explicitly noting the short-term and acute nature of previous investigations (Lines 11-12).

In the concluding sentence, “healthier fluid intake” is a stretch, given that total sugar-sweetened beverage intake did not change. Moreover, one should not repeat results. It’s better to say that a moderate increase in plain water intake may prompt a decrease in daily caloric intake and thus facilitate weight management.

We thank the reviewer for this important comment and agree that the phrase “healthier fluid intake” was overly broad and not fully supported by our findings, particularly given the lack of change in sugar-sweetened beverage intake. We have revised the concluding statement to more accurately reflect the observed outcomes and avoid overgeneralization (Lines 31-32).

Introduction

At the end of the second paragraph, change to read “…requirements and individualization (i.e., age, SEX, physical activity level, health status,…”

Revised as suggested.

In the last paragraph, the authors state “Although the relationship between water consumption and energy intake has been examined…” while citing numerous prior publications. Emphasize more specifically the shortcoming of these prior investigations (short-term, cross-sectional?) and explicitly why this study is important. Just because “less is known about a longitudinal intervention” does not then translate into “Therefore, it is critical to investigate the relationship between water consumption and fluid and calorie intake.” Avoid getting into specific methods here (move the description of the two 500-ml water bottles to the Methods). However, state a clear measurable hypothesis and emphatically state why this study and anticipated findings are important. Compel the reader to want to explore further!

We thank the reviewer for this insightful and constructive comment. The final paragraph of the Introduction has been revised (Lines 75–86) to better highlight the limitations of prior research, including the predominance of short-term and cross-sectional designs and the focus on overweight or obese populations. Methodological details have been removed to maintain a clear distinction between the Introduction and Methods sections. Furthermore, the rationale for the study has been strengthened, and a clear, testable hypothesis has been added.

Methods

Right away in the Methods, need to better clarify that the I L of plain water was in addition to their inherent self-determined habitual fluid intake prior to the intervention.

Clarify when the additional plain water intake began. Does the first (baseline) 24-hour collection of urine and all fluids/foods include the additional water? One cannot fully interpret the results without clarification on this.

We thank the reviewer for these important comments. We have revised the Methods section (Lines 113–128) to clearly state that the prescribed 1 L/day of plain water was consumed in addition to participants’ habitual, self-determined fluid intake. We have also clarified the timing of the intervention, specifying that baseline measurements (including the initial 24-hour urine and dietary/fluid intake assessments) were collected prior to the initiation of the additional water intake. The prescribed water consumption began only after completion of baseline assessments. These clarifications ensure accurate interpretation of the changes observed across time points.

In the Assessment of 24-hour Fluid, Plain water, Sugar-sweetened Beverages, and Caloric

Intake section, be consistent with the utility of i.e., meaning that is and e.g., meaning for example, which are also missing in some places.

We thank the reviewer for this helpful comment. We have carefully reviewed the manuscript and revised the Assessment of 24-hour Fluid, Plain Water, Sugar-Sweetened Beverages, and Caloric Intake section (Lines 131–150) to ensure consistent and correct usage of i.e. (that is) and e.g. (for example). Missing instances have been added where appropriate, and incorrect usages have been corrected throughout the section.

Indicate if/how the urine collection container tare weight was accounted for.

We thank the reviewer for this important clarification. We have revised the Methods section (Lines 158–160) to explicitly state that the tare weight of the urine collection container was measured prior to distribution and subtracted from the total recorded weight to obtain the net urine volume. This ensures accurate quantification of 24-hour urine output.

Discussion

Succinct technical writing using active voice is preferable: please reword the first 2 sentences to read “Our study investigated the effect of a six-week intervention… which revealed several important findings.”

We thank the reviewer for this suggestion. We have revised the opening sentences of the Discussion to improve clarity and adopt a more concise, active voice style, as recommended (Lines 217–219).

The increase in 24-h total fluid intake was temporary. Reword this statement in the first paragraph to indicate an interim increase in total fluid intake with a more lasting change in greater plain water intake.

We thank the reviewer for this helpful suggestion. We have revised the statement in the Discussion (Lines 230-235) to more clearly distinguish the transient increase in total fluid intake from the more sustained increase in plain water consumption.

The lack of statistical change in USG or Uosm speak more to the variability of these measures (and influencing factors) vs. the sensitivity in indicating changes in hydration status. Numerous studies support this and should be noted.

We thank the reviewer for this important insight. We have revised the Discussion section (Lines 328–335) to clarify that the lack of significant changes in USG and U_osm_ may reflect the inherent variability of these biomarkers and their susceptibility to external influencing factors, rather than a true absence of physiological response. We also incorporated supporting literature to highlight the limitations and sensitivity of urinary hydration biomarkers in detecting subtle changes in hydration status, particularly in free-living, healthy populations.

The last sentence of the Limitations and Future Study section repeats itself. Proofread.

We thank the reviewer for this observation. The redundant sentence has been removed to improve clarity and conciseness.

Most of the Conclusions section repeats what is already stated. Focus and expand on the last sentence from a translation and practical applications/utility perspective.

We thank the reviewer for this valuable suggestion. The Conclusions section has been revised to reduce redundancy and to more clearly emphasize the translational and practical implications of the findings, with particular attention to the final statement (Lines 384-395).

Reviewer #3: This manuscript by Zhao et al investigates prescribed 1L of fluid per day and the impact on nutritional intake in young healthy males and females.

General comments:

For the 24-h urine collection, were all samples collected in the jug, or were first morning samples collected separately (to evaluate first morning color, USG, osmolality) and then added to the jug?

Thank you for this important clarification. In the present study, all urine samples were collected as part of a continuous 24-hour collection into two urine jugs. Participants were instructed to collect all urine voids over the 24-hour period in the provided container. We did not collect or analyze first morning urine samples separately, nor did we assess first morning urine color, specific gravity (USG), or osmolality independently. Accordingly, all hydration-related urine biomarkers reported in this study reflect the integrated 24-hour urine sample rather than discrete time-specific (e.g., first morning) measurements.

This clarification has been added to the Methods section to ensure consistency and improve interpretability of the hydration status outcomes (Lines 153-156).

Discussion: The authors report benefits to the 1L prescribed fluid per day, but the volunteers were already hydrated prior to the intervention (per USG) and there was no change to their hydration status or caloric intake throughout the 6-week intervention period. Thus, is the prescribed water beneficial?

We thank the reviewer for this insightful comment. Although participants were generally euhydrated at baseline, the intervention successfully increased total fluid intake and improved adherence to National Academy of Medicine (NAM) guidelines. Importantly, the primary utility of the prescribed 1 L/day fluid intervention may lie in its ability to promote compliance with recommended fluid intake levels and to increase plain water consumption, rather than in inducing further changes in hydration status among individuals who are already euhydrated. We have revised the manuscript to clarify this interpretation and to better emphasize the practical significance of the intervention (Lines 245-255).

This reviewer would recommend more robust statistical methods including mixed effects models to allow for greater control relative to ANOVA.

We thank the reviewer for this valuable suggestion. We agree that mixed-effects models provide a more robust framework for analyzing repeated-measures data due to their ability to account for within-subject variability and missing data. We would like to clarify that a mixed-effects model was in fact used for the primary analyses in this study. We apologize for the lack of clarity in the original manuscript, where the statistical approach was incorrectly described as a one-way repeated measures ANOVA.

We have revised the Methods section (Lines 174-176) to accurately reflect the use of mixed-effects modeling and have clarified the statistical approach throughout the manuscript. The results and conclusions remain unchanged.

Can the authors define the volunteers habitual fluid intake pattern? Was this measured? If not, how can the authors suggest that the increase in fluid intake at the midpoint but not post was related to habitual fluid intake?

We thank the reviewer for this important comment. Habitual fluid intake patterns were not directly quantified beyond the baseline 24-hour fluid intake assessment. Therefore, we cannot definitively attribute the observed increase in total fluid intake at mid but not post to a return to habitual intake patterns. We have revised the manuscript to clarify this point and avoid overinterpretation (Lines 230-240). Specifically, we now interpret the attenuation in total fluid intake more conservatively as a potential combination of behavioral adaptation, compensatory reduction in other beverages, or variability in adherence over time.

Can the authors provide more clarity on the following statement: “Clinically, however, the increase of 238mL in 24-hour total fluid intake after the intervention may still contribute to long-term health benefits.” There was no effect at post following the intervention. Additionally, if the volunteers were already hydrated, what benefits would they experience? Please clarify.

We appreciate the reviewer’s comment. We agree that the clinical relevance of the 238 mL increase should be interpreted with caution, particularly given that the effect was not sustained at post-intervention and participants were already euhydrated at baseline. We have revised the manuscript to clarify that this modest and transient increase is unlikely to confer meaningful hydration-related benefits in an already hydrated population (Lines 240-243). Instead, we emphasize that the primary observed benefit of the intervention was behavioral, namely, an increase in plain water consumption and improved adherence to fluid intake recommendations rather than physiological changes in hydration status.

Can the authors do a sub analysis on individuals who were more dehydrated (assuming there was a spectrum in the n=38)? This reviewer would hypothesize that individuals who were more dehydrated to start may have garnered a larger benefit, and these data could be more impactful in that context.

We thank the reviewer for this insightful suggestion. We agree that examining whether individuals with lower baseline hydration status respond differently to the intervention is an important and interesting consideration. However, in the present study, participants were generally euhydrated at baseline based on both USG and U_osm_, with only a small proportion falling outside established hydration thresholds. As such, any subgroup analysis based on baseline hydration status would result in very small group sizes, limiting statistical power and increasing the likelihood of misleading or non-generalizable findings.

We have added clarification in the manuscript to acknowledge this limitation and to suggest that future studies specifically recruit participants across a broader range of hydration statuses to better address this question (Lines359-368).

Can the authors also contextualize the NAM guidelines? Particularly given the volunteers all had USG suggesting euhydrated status. It is clear the intervention increased those that met the guidelines, but to what extent, given the sample.

We thank the reviewer for this helpful comment. The National Academy of Medicine (NAM) guidelines represent population-level adequate intake values derived from national survey data and are intended as general recommendations rather than individualized hydration targets. In the present study, although the proportion of participants meeting NAM guidelines increased from 3.1% at baseline to 18.8% at post-intervention, the majority of participants remained below these intake thresholds. Importantly, participants were already euhydrated at baseline based on USG and Uosm, indicating that meeting NAM guidelines was not necessary to achieve adequate hydration status in this sample.

Therefore, while the intervention improved adherence to guideline-based intake recommendations, the magnitude of change was modest and did not translate to measurable physiological changes in hydration status. We have revised the manuscript to better contextualize these findings and clarify that NAM guidelines should be interpreted alongside individual hydration markers (Line 245-255).

Minor comments:

Typo in the statistical analysis section “((N>38)”

We thank the reviewer for identifying this typographical error. The error has been corrected in the revised manuscript.
