# Supplementary material for: Effect of intentional daily liter of plain water on total fluid and caloric intake among free-living adults
Source: PLoS One. 2026 Jul 21;21(7):e0352203. doi: 10.1371/journal.pone.0352203 (PMC13387533; doi:10.1371/journal.pone.0352203)
Supplement: Peer review history

# Peer review: Decision Letter 0

**DOI:** 10.1371/journal.pone.0352203.r001

**Authors:** William Adams

6 Apr 2026

PONE-D-26-07092

Effect of Intentional Daily Liter of Plain Water on Fluid and Caloric Intake among Free-living Adults

PLOS One

Dear Dr. McDermott,

Thank you for submitting your manuscript to PLOS ONE. After careful consideration, we feel that it has merit but does not fully meet PLOS ONE’s publication criteria as it currently stands. Therefore, we invite you to submit a revised version of the manuscript that addresses the points raised during the review process.

Please submit your revised manuscript by May 21 2026 11:59PM. If you will need more time than this to complete your revisions, please reply to this message or contact the journal office at plosone@plos.org. When you're ready to submit your revision, log on to https://www.editorialmanager.com/pone/ and select the 'Submissions Needing Revision' folder to locate your manuscript file.

Please include the following items when submitting your revised manuscript:

- A letter that responds to each point raised by the academic editor and reviewer(s). You should upload this letter as a separate file labeled 'Response to Reviewers'.
- A marked-up copy of your manuscript that highlights changes made to the original version. You should upload this as a separate file labeled 'Revised Manuscript with Track Changes'.
- An unmarked version of your revised paper without tracked changes. You should upload this as a separate file labeled 'Manuscript'.

If you would like to make changes to your financial disclosure, please include your updated statement in your cover letter. Guidelines for resubmitting your figure files are available below the reviewer comments at the end of this letter.

If applicable, we recommend that you deposit your laboratory protocols in protocols.io to enhance the reproducibility of your results. Protocols.io assigns your protocol its own identifier (DOI) so that it can be cited independently in the future. For instructions see: https://journals.plos.org/plosone/s/submission-guidelines#loc-laboratory-protocols. Additionally, PLOS ONE offers an option for publishing peer-reviewed Lab Protocol articles, which describe protocols hosted on protocols.io. Read more information on sharing protocols at https://plos.org/protocols?utm_medium=editorial-email&utm_source=authorletters&utm_campaign=protocols.

As the corresponding author, your ORCID iD is verified in the submission system and will appear in the published article. PLOS supports the use of ORCID, and we encourage all coauthors to register for an ORCID iD and use it as well. Please encourage your coauthors to verify their ORCID iD within the submission system before final acceptance, as unverified ORCID iDs will not appear in the published article. *Only*  the individual author can complete the verification step; PLOS staff *cannot*  verify ORCID iDs on behalf of authors.

We look forward to receiving your revised manuscript.

Kind regards,

William M. Adams

Academic Editor

PLOS One

Journal Requirements:

When submitting your revision, we need you to address these additional requirements.

1. Please ensure that your manuscript meets PLOS ONE's style requirements, including those for file naming. The PLOS ONE style templates can be found at

https://journals.plos.org/plosone/s/file?id=wjVg/PLOSOne_formatting_sample_main_body.pdf and

https://journals.plos.org/plosone/s/file?id=ba62/PLOSOne_formatting_sample_title_authors_affiliations.pdf

2. We noticed you have some minor occurrence of overlapping text with the following previous publication(s), which needs to be addressed:

https://www.mdpi.com/2072-6643/16/11/1541

In your revision ensure you cite all your sources (including your own works), and quote or rephrase any duplicated text outside the methods section. Further consideration is dependent on these concerns being addressed.

3. Thank you for stating the following financial disclosure:

"This research was supported by the Chancellor’s Innovation Fund at the University of Arkansas."

Please state what role the funders took in the study.  If the funders had no role, please state: "The funders had no role in study design, data collection and analysis, decision to publish, or preparation of the manuscript."

If this statement is not correct you must amend it as needed.

Please include this amended Role of Funder statement in your cover letter; we will change the online submission form on your behalf.

4. In this instance it seems there may be acceptable restrictions in place that prevent the public sharing of your minimal data. However, in line with our goal of ensuring long-term data availability to all interested researchers, PLOS’ Data Policy states that authors cannot be the sole named individuals responsible for ensuring data access (http://journals.plos.org/plosone/s/data-availability#loc-acceptable-data-sharing-methods).

Data requests to a non-author institutional point of contact, such as a data access or ethics committee, helps guarantee long term stability and availability of data. Providing interested researchers with a durable point of contact ensures data will be accessible even if an author changes email addresses, institutions, or becomes unavailable to answer requests.

Before we proceed with your manuscript, please also provide non-author contact information (phone/email/hyperlink) for a data access committee, ethics committee, or other institutional body to which data requests may be sent. If no institutional body is available to respond to requests for your minimal data, please consider if there any institutional representatives who did not collaborate in the study, and are not listed as authors on the manuscript, who would be able to hold the data and respond to external requests for data access? If so, please provide their contact information (i.e., email address). Please also provide details on how you will ensure persistent or long-term data storage and availability.

If the reviewer comments include a recommendation to cite specific previously published works, please review and evaluate these publications to determine whether they are relevant and should be cited. There is no requirement to cite these works unless the editor has indicated otherwise.

[Note: HTML markup is below. Please do not edit.]

Reviewers' comments:

Reviewer's Responses to Questions

**Comments to the Author**

1. Is the manuscript technically sound, and do the data support the conclusions?

The manuscript must describe a technically sound piece of scientific research with data that supports the conclusions. Experiments must have been conducted rigorously, with appropriate controls, replication, and sample sizes. The conclusions must be drawn appropriately based on the data presented.

Reviewer #1: Yes

Reviewer #2: Yes

Reviewer #3: Partly

**********

2. Has the statistical analysis been performed appropriately and rigorously?

Reviewer #1: No

Reviewer #2: Yes

Reviewer #3: No

**********

3. Have the authors made all data underlying the findings in their manuscript fully available?

The PLOS Data policy requires authors to make all data underlying the findings described in their manuscript fully available without restriction, with rare exception (please refer to the Data Availability Statement in the manuscript PDF file). The data should be provided as part of the manuscript or its supporting information, or deposited to a public repository. For example, in addition to summary statistics, the data points behind means, medians and variance measures should be available. If there are restrictions on publicly sharing data—e.g. participant privacy or use of data from a third party—those must be specified.

Reviewer #1: Yes

Reviewer #2: Yes

Reviewer #3: Yes

**********

4. Is the manuscript presented in an intelligible fashion and written in standard English?

PLOS ONE does not copyedit accepted manuscripts, so the language in submitted articles must be clear, correct, and unambiguous. Any typographical or grammatical errors should be corrected at revision, so please note any specific errors here.

Reviewer #1: Yes

Reviewer #2: Yes

Reviewer #3: Yes

**********

5. Review Comments to the Author

Please use the space provided to explain your answers to the questions above. You may also include additional comments for the author, including concerns about dual publication, research ethics, or publication ethics. (Please upload your review as an attachment if it exceeds 20,000 characters)

Reviewer #1: Introduction

What is the rational to study this population “less is known about the effects of a longitudinal intervention in non-obese, young adults.”?

Method

“All participants self-identified as generally healthy.” is too broad.

“1 liter of plain water daily for six weeks” means additional 1L to what they normally do? More information is needed in general. What was the instruction? Methods and procedures might not be enough.

Did participants know this was hydration study? If so, that typically impacts fluid intake as participants tend to increase water intake by knowing this was assessing hydration.

“Pearson correlation was used to investigate the relationship between 24-hour total caloric intake and total fluid intake, total plain water intake, and total sugar-sweetened beverages intake.” Since multiple time points are included from one participant, repeated correlation is better.

Discussion

“This pattern indicates the intervention may have temporarily promoted greater fluid intake, which diminished over time as participants returned toward their habitual fluid intake pattern.” means participants decreased the amount of water intake outside of additional 1 L from mid to post. Does this indicate the intervention was not successful? Please clarify!

“Consistent with this notion, previous studies have demonstrated that water consumption enhances satiety and decreases subsequent energy intake, thereby contributing to weight loss [27,28, 41].” needs to be carefully interpreted as fluid intake was not increased at post.

Reviewer #2: Specific Comments (Suggested edits appear in CAPS)

Title: Should read “…TOTAL Fluid and Caloric Intake…”

Abstract

To clarify the contrast, the first sentence should read (or similar) “While previous studies have examined SHORT-TERM water consumption and CORRESPONDING energy intake…”

In the concluding sentence, “healthier fluid intake” is a stretch, given that total sugar-sweetened beverage intake did not change. Moreover, one should not repeat results. It’s better to say that a moderate increase in plain water intake may prompt a decrease in daily caloric intake and thus facilitate weight management.

Introduction

At the end of the second paragraph, change to read “…requirements and individualization (i.e., age, SEX, physical activity level, health status,…”

In the last paragraph, the authors state “Although the relationship between water consumption and energy intake has been examined…” while citing numerous prior publications. Emphasize more specifically the shortcoming of these prior investigations (short-term, cross-sectional?) and explicitly why this study is important. Just because “less is known about a longitudinal intervention” does not then translate into “Therefore, it is critical to investigate the relationship between water consumption and fluid and calorie intake.” Avoid getting into specific methods here (move the description of the two 500-ml water bottles to the Methods). However, state a clear measurable hypothesis and emphatically state why this study and anticipated findings are important. Compel the reader to want to explore further!

Methods

Right away in the Methods, need to better clarify that the I L of plain water was in addition to their inherent self-determined habitual fluid intake prior to the intervention.

Clarify when the additional plain water intake began. Does the first (baseline) 24-hour collection of urine and all fluids/foods include the additional water? One cannot fully interpret the results without clarification on this.

In the Assessment of 24-hour Fluid, Plain water, Sugar-sweetened Beverages, and Caloric

Intake section, be consistent with the utility of i.e., meaning that is and e.g., meaning for example, which are also missing in some places.

Indicate if/how the urine collection container tare weight was accounted for.

Discussion

Succinct technical writing using active voice is preferable: please reword the first 2 sentences to read “Our study investigated the effect of a six-week intervention… which revealed several important findings.”

The increase in 24-h total fluid intake was temporary. Reword this statement in the first paragraph to indicate an interim increase in total fluid intake with a more lasting change in greater plain water intake.

The lack of statistical change in USG or Uosm speak more to the variability of these measures (and influencing factors) vs. the sensitivity in indicating changes in hydration status. Numerous studies support this and should be noted.

The last sentence of the Limitations and Future Study section repeats itself. Proofread.

Most of the Conclusions section repeats what is already stated. Focus and expand on the last sentence from a translation and practical applications/utility perspective.

Reviewer #3: This manuscript by Zhao et al investigates prescribed 1L of fluid per day and the impact on nutritional intake in young healthy males and females.

General comments:

For the 24-h urine collection, were all samples collected in the jug, or were first morning samples collected separately (to evaluate first morning color, USG, osmolality) and then added to the jug?

Discussion: The authors report benefits to the 1L prescribed fluid per day, but the volunteers were already hydrated prior to the intervention (per USG) and there was no change to their hydration status or caloric intake throughout the 6-week intervention period. Thus, is the prescribed water beneficial?

This reviewer would recommend more robust statistical methods including mixed effects models to allow for greater control relative to ANOVA.

Can the authors define the volunteers habitual fluid intake pattern? Was this measured? If not, how can the authors suggest that the increase in fluid intake at the midpoint but not post was related to habitual fluid intake?

Can the authors provide more clarity on the following statement: “Clinically, however, the increase of 238mL in 24-hour total fluid intake after the intervention may still contribute to long-term health benefits.” There was no effect at post following the intervention. Additionally, if the volunteers were already hydrated, what benefits would they experience? Please clarify.

Can the authors do a sub analysis on individuals who were more dehydrated (assuming there was a spectrum in the n=38)? This reviewer would hypothesize that individuals who were more dehydrated to start may have garnered a larger benefit, and these data could be more impactful in that context.

Can the authors also contextualize the NAM guidelines? Particularly given the volunteers all had USG suggesting euhydrated status. It is clear the intervention increased those that met the guidelines, but to what extent, given the sample.

Minor comments:

Typo in the statistical analysis section “((N>38)”

**********

6. PLOS authors have the option to publish the peer review history of their article (what does this mean?). If published, this will include your full peer review and any attached files.

If you choose “no”, your identity will remain anonymous but your review may still be made public.

**Do you want your identity to be public for this peer review?**  For information about this choice, including consent withdrawal, please see our Privacy Policy.

Reviewer #1: No

Reviewer #2: No

Reviewer #3: No

**********

[NOTE: If reviewer comments were submitted as an attachment file, they will be attached to this email and accessible via the submission site. Please log into your account, locate the manuscript record, and check for the action link "View Attachments". If this link does not appear, there are no attachment files.]

To ensure your figures meet our technical requirements, please review our figure guidelines: https://journals.plos.org/plosone/s/figures

You may also use PLOS’s free figure tool, NAAS, to help you prepare publication quality figures: https://journals.plos.org/plosone/s/figures#loc-tools-for-figure-preparation.

NAAS will assess whether your figures meet our technical requirements by comparing each figure against our figure specifications.

# Author comment: Author response to Decision Letter 1

**DOI:** 10.1371/journal.pone.0352203.r002

12 May 2026

Review Comments to the Author

Please use the space provided to explain your answers to the questions above. You may also include additional comments for the author, including concerns about dual publication, research ethics, or publication ethics. (Please upload your review as an attachment if it exceeds 20,000 characters)

Reviewer #1: Introduction

What is the rational to study this population “less is known about the effects of a longitudinal intervention in non-obese, young adults.”?

We thank the reviewer for this important comment. We have strengthened the rationale by emphasizing the limited longitudinal evidence in healthy, non-obese populations and the importance of preventive behavioral strategies (Lines 75-82).

Method

“All participants self-identified as generally healthy.” is too broad.

We thank the reviewer for this comment and agree that the phrasing was overly broad. We have revised the description to clarify that participant health status was determined based on predefined inclusion criteria, including absence of diagnosed chronic medical conditions and use of medications known to affect fluid balance (Lines 98-101). The phrase “self-identified as generally healthy” has been removed to avoid ambiguity.

“1 liter of plain water daily for six weeks” means additional 1L to what they normally do? More information is needed in general. What was the instruction? Methods and procedures might not be enough.

We thank the reviewer for this important comment and agree that additional clarification of the intervention protocol is needed. We have revised the Methods section to explicitly state that participants were instructed to consume 1 L/day of plain water in addition to their habitual fluid intake. We also expanded the description of the instructions provided to participants, including how the water intake was distributed and how participants were asked to maintain their usual dietary and fluid consumption behaviors outside of the prescribed intervention (Lines 113-124).

Did participants know this was hydration study? If so, that typically impacts fluid intake as participants tend to increase water intake by knowing this was assessing hydration.

We thank the reviewer for this important observation. Participants were informed that the study involved fluid intake and hydration-related behaviors as part of the consent process. We acknowledge that this awareness may have influenced participants’ habitual fluid intake (e.g., increasing water consumption independent of the prescribed intervention). To address this, we have clarified this point in the Methods section (Lines 125-128) and acknowledged it as a potential source of bias in the Discussion (Lines 353-357).

“Pearson correlation was used to investigate the relationship between 24-hour total caloric intake and total fluid intake, total plain water intake, and total sugar-sweetened beverages intake.” Since multiple time points are included from one participant, repeated correlation is better.

We agree with the reviewer that a Pearson correlation does not account for the non-independence of repeated measures data. Accordingly, we have re-analyzed the relationships between 24-hour caloric intake and fluid variables using repeated measures correlation. The Results section have been updated to reflect these more robust statistical findings (Lines 178-182).

Discussion

“This pattern indicates the intervention may have temporarily promoted greater fluid intake, which diminished over time as participants returned toward their habitual fluid intake pattern.” means participants decreased the amount of water intake outside of additional 1 L from mid to post. Does this indicate the intervention was not successful? Please clarify!

We thank the reviewer for this insightful comment. We agree that the original wording may have implied ambiguity regarding the effectiveness of the intervention. We have revised the Discussion to clarify that the intervention was partially successful (Lines 230-238), specifically, it increased fluid intake during the early phase of the intervention, but this effect was not fully sustained over time. We further clarify that the attenuation in total fluid intake likely reflects behavioral adaptation or reduced adherence rather than complete intervention failure.

“Consistent with this notion, previous studies have demonstrated that water consumption enhances satiety and decreases subsequent energy intake, thereby contributing to weight loss [27,28, 41].” needs to be carefully interpreted as fluid intake was not increased at post.

We thank the reviewer for this important observation and agree that the interpretation of prior literature should be better aligned with our findings. We have revised the Discussion to avoid overgeneralization and to clarify that the proposed mechanisms linking water intake to reduced energy intake were not consistently supported in our study, particularly at the post-intervention time point (Lines 285-290).

Reviewer #2: Specific Comments (Suggested edits appear in CAPS)

Title: Should read “…TOTAL Fluid and Caloric Intake…”

Revised as suggested.

Abstract

To clarify the contrast, the first sentence should read (or similar) “While previous studies have examined SHORT-TERM water consumption and CORRESPONDING energy intake…”

We thank the reviewer for this helpful suggestion. We have revised the opening sentence of the Abstract to better emphasize the contrast with prior literature by explicitly noting the short-term and acute nature of previous investigations (Lines 11-12).

In the concluding sentence, “healthier fluid intake” is a stretch, given that total sugar-sweetened beverage intake did not change. Moreover, one should not repeat results. It’s better to say that a moderate increase in plain water intake may prompt a decrease in daily caloric intake and thus facilitate weight management.

We thank the reviewer for this important comment and agree that the phrase “healthier fluid intake” was overly broad and not fully supported by our findings, particularly given the lack of change in sugar-sweetened beverage intake. We have revised the concluding statement to more accurately reflect the observed outcomes and avoid overgeneralization (Lines 31-32).

Introduction

At the end of the second paragraph, change to read “…requirements and individualization (i.e., age, SEX, physical activity level, health status,…”

Revised as suggested.

In the last paragraph, the authors state “Although the relationship between water consumption and energy intake has been examined…” while citing numerous prior publications. Emphasize more specifically the shortcoming of these prior investigations (short-term, cross-sectional?) and explicitly why this study is important. Just because “less is known about a longitudinal intervention” does not then translate into “Therefore, it is critical to investigate the relationship between water consumption and fluid and calorie intake.” Avoid getting into specific methods here (move the description of the two 500-ml water bottles to the Methods). However, state a clear measurable hypothesis and emphatically state why this study and anticipated findings are important. Compel the reader to want to explore further!

We thank the reviewer for this insightful and constructive comment. The final paragraph of the Introduction has been revised (Lines 75–86) to better highlight the limitations of prior research, including the predominance of short-term and cross-sectional designs and the focus on overweight or obese populations. Methodological details have been removed to maintain a clear distinction between the Introduction and Methods sections. Furthermore, the rationale for the study has been strengthened, and a clear, testable hypothesis has been added.

Methods

Right away in the Methods, need to better clarify that the I L of plain water was in addition to their inherent self-determined habitual fluid intake prior to the intervention.

Clarify when the additional plain water intake began. Does the first (baseline) 24-hour collection of urine and all fluids/foods include the additional water? One cannot fully interpret the results without clarification on this.

We thank the reviewer for these important comments. We have revised the Methods section (Lines 113–128) to clearly state that the prescribed 1 L/day of plain water was consumed in addition to participants’ habitual, self-determined fluid intake. We have also clarified the timing of the intervention, specifying that baseline measurements (including the initial 24-hour urine and dietary/fluid intake assessments) were collected prior to the initiation of the additional water intake. The prescribed water consumption began only after completion of baseline assessments. These clarifications ensure accurate interpretation of the changes observed across time points.

In the Assessment of 24-hour Fluid, Plain water, Sugar-sweetened Beverages, and Caloric

Intake section, be consistent with the utility of i.e., meaning that is and e.g., meaning for example, which are also missing in some places.

We thank the reviewer for this helpful comment. We have carefully reviewed the manuscript and revised the Assessment of 24-hour Fluid, Plain Water, Sugar-Sweetened Beverages, and Caloric Intake section (Lines 131–150) to ensure consistent and correct usage of i.e. (that is) and e.g. (for example). Missing instances have been added where appropriate, and incorrect usages have been corrected throughout the section.

Indicate if/how the urine collection container tare weight was accounted for.

We thank the reviewer for this important clarification. We have revised the Methods section (Lines 158–160) to explicitly state that the tare weight of the urine collection container was measured prior to distribution and subtracted from the total recorded weight to obtain the net urine volume. This ensures accurate quantification of 24-hour urine output.

Discussion

Succinct technical writing using active voice is preferable: please reword the first 2 sentences to read “Our study investigated the effect of a six-week intervention… which revealed several important findings.”

We thank the reviewer for this suggestion. We have revised the opening sentences of the Discussion to improve clarity and adopt a more concise, active voice style, as recommended (Lines 217–219).

The increase in 24-h total fluid intake was temporary. Reword this statement in the first paragraph to indicate an interim increase in total fluid intake with a more lasting change in greater plain water intake.

We thank the reviewer for this helpful suggestion. We have revised the statement in the Discussion (Lines 230-235) to more clearly distinguish the transient increase in total fluid intake from the more sustained increase in plain water consumption.

The lack of statistical change in USG or Uosm speak more to the variability of these measures (and influencing factors) vs. the sensitivity in indicating changes in hydration status. Numerous studies support this and should be noted.

We thank the reviewer for this important insight. We have revised the Discussion section (Lines 328–335) to clarify that the lack of significant changes in USG and Uosm may reflect the inherent variability of these biomarkers and their susceptibility to external influencing factors, rather than a true absence of physiological response. We also incorporated supporting literature to highlight the limitations and sensitivity of urinary hydration biomarkers in detecting subtle changes in hydration status, particularly in free-living, healthy populations.

The last sentence of the Limitations and Future Study section repeats itself. Proofread.

We thank the reviewer for this observation. The redundant sentence has been removed to improve clarity and conciseness.

Most of the Conclusions section repeats what is already stated. Focus and expand on the last sentence from a translation and practical applications/utility perspective.

We thank the reviewer for this valuable suggestion. The Conclusions section has been revised to reduce redundancy and to more clearly emphasize the translational and practical implications of the findings, with particular attention to the final statement (Lines 384-395).

Reviewer #3: This manuscript by Zhao et al investigates prescribed 1L of fluid per day and the impact on nutritional intake in young healthy males and females.

General comments:

For the 24-h urine collection, were all samples collected in the jug, or were first morning samples collected separately (to evaluate first morning color, USG, osmolality) and then added to the jug?

Thank you for this important clarification. In the present study, all urine samples were collected as part of a continuous 24-hour collection into two urine jugs. Participants were instructed to collect all urine voids over the 24-hour period in the provided container. We did not collect or analyze first morning urine samples separately, nor did we assess first morning urine color, specific gravity (USG), or osmolality independently. Accordingly, all hydration-related urine biomarkers reported in this study reflect the integrated 24-hour urine sample rather than discrete time-specific (e.g., first morning) measurements.

This clarification has been added to the Methods section to ensure consistency and improve interpretability of the hydration status outcomes (Lines 153-156).

Discussion: The authors report benefits to the 1L prescribed fluid per day, but the volunteers were already hydrated prior to the intervention (per USG) and there was no change to their hydration status or caloric intake throughout the 6-week intervention period. Thus, is the prescribed water beneficial?

We thank the reviewer for this insightful comment. Although participants were generally euhydrated at baseline, the intervention successfully increased total fluid intake and improved adherence to National Academy of Medicine (NAM) guidelines. Importantly, the primary utility of the prescribed 1 L/day fluid intervention may lie in its ability to promote compliance with recommended fluid intake levels and to increase plain water consumption, rather than in inducing further changes in hydration status among individuals who are already euhydrated. We have revised the manuscript to clarify this interpretation and to better emphasize the practical significance of the intervention (Lines 245-255).

This reviewer would recommend more robust statistical methods including mixed effects models to allow for greater control relative to ANOVA.

We thank the reviewer for this valuable suggestion. We agree that mixed-effects models provide a more robust framework for analyzing repeated-measures data due to their ability to account for within-subject variability and missing data. We would like to clarify that a mixed-effects model was in fact used for the primary analyses in this study. We apologize for the lack of clarity in the original manuscript, where the statistical approach was incorrectly described as a one-way repeated measures ANOVA.

We have revised the Methods section (Lines 174-176) to accurately reflect the use of mixed-effects modeling and have clarified the statistical approach throughout the manuscript. The results and conclusions remain unchanged.

Can the authors define the volunteers habitual fluid intake pattern? Was this measured? If not, how can the authors suggest that the increase in fluid intake at the midpoint but not post was related to habitual fluid intake?

We thank the reviewer for this important comment. Habitual fluid intake patterns were not directly quantified beyond the baseline 24-hour fluid intake assessment. Therefore, we cannot definitively attribute the observed increase in total fluid intake at mid but not post to a return to habitual intake patterns. We have revised the manuscript to clarify this point and avoid overinterpretation (Lines 230-240). Specifically, we now interpret the attenuation in total fluid intake more conservatively as a potential combination of behavioral adaptation, compensatory reduction in other beverages, or variability in adh

Attachment Submitted filename: Responses to reviewerss comments_PLOS One_R1.docx

# Peer review: Decision Letter 1

**DOI:** 10.1371/journal.pone.0352203.r003

**Authors:** William Adams

7 Jun 2026

Effect of Intentional Daily Liter of Plain Water on Fluid and Caloric Intake among Free-living Adults

PONE-D-26-07092R1

Dear Dr. McDermott,

We’re pleased to inform you that your manuscript has been judged scientifically suitable for publication and will be formally accepted for publication once it meets all outstanding technical requirements.

Within one week, you’ll receive an e-mail detailing the required amendments. When these have been addressed, you’ll receive a formal acceptance letter and your manuscript will be scheduled for publication.

An invoice will be generated when your article is formally accepted. Please note, if your institution has a publishing partnership with PLOS and your article meets the relevant criteria, all or part of your publication costs will be covered. Please make sure your user information is up-to-date by logging into Editorial Manager at Editorial Manager® and clicking the ‘Update My Information' link at the top of the page. For questions related to billing, please contact billing support.

If your institution or institutions have a press office, please notify them about your upcoming paper to help maximize its impact. If they’ll be preparing press materials, please inform our press team as soon as possible -- no later than 48 hours after receiving the formal acceptance. Your manuscript will remain under strict press embargo until 2 pm Eastern Time on the date of publication. For more information, please contact onepress@plos.org.

Kind regards,

William M. Adams

Academic Editor

PLOS One

Additional Editor Comments (optional):

Reviewers' comments:

Reviewer's Responses to Questions

**Comments to the Author**

1. If the authors have adequately addressed your comments raised in a previous round of review and you feel that this manuscript is now acceptable for publication, you may indicate that here to bypass the “Comments to the Author” section, enter your conflict of interest statement in the “Confidential to Editor” section, and submit your "Accept" recommendation.

Reviewer #1: All comments have been addressed

Reviewer #2: (No Response)

Reviewer #3: All comments have been addressed

**********

2. Is the manuscript technically sound, and do the data support the conclusions?

The manuscript must describe a technically sound piece of scientific research with data that supports the conclusions. Experiments must have been conducted rigorously, with appropriate controls, replication, and sample sizes. The conclusions must be drawn appropriately based on the data presented.

Reviewer #1: Yes

Reviewer #2: Yes

Reviewer #3: Yes

**********

3. Has the statistical analysis been performed appropriately and rigorously?

Reviewer #1: Yes

Reviewer #2: Yes

Reviewer #3: Yes

**********

4. Have the authors made all data underlying the findings in their manuscript fully available?

The PLOS Data policy requires authors to make all data underlying the findings described in their manuscript fully available without restriction, with rare exception (please refer to the Data Availability Statement in the manuscript PDF file). The data should be provided as part of the manuscript or its supporting information, or deposited to a public repository. For example, in addition to summary statistics, the data points behind means, medians and variance measures should be available. If there are restrictions on publicly sharing data—e.g. participant privacy or use of data from a third party—those must be specified.

Reviewer #1: Yes

Reviewer #2: Yes

Reviewer #3: Yes

**********

5. Is the manuscript presented in an intelligible fashion and written in standard English?

PLOS ONE does not copyedit accepted manuscripts, so the language in submitted articles must be clear, correct, and unambiguous. Any typographical or grammatical errors should be corrected at revision, so please note any specific errors here.

Reviewer #1: Yes

Reviewer #2: Yes

Reviewer #3: Yes

**********

6. Review Comments to the Author

Please use the space provided to explain your answers to the questions above. You may also include additional comments for the author, including concerns about dual publication, research ethics, or publication ethics. (Please upload your review as an attachment if it exceeds 20,000 characters)

Reviewer #1: Thank you for addressing all comments. The paper improved a lot and it is very interesting. It is also addressing an important question.

Reviewer #2: All of my suggested edits and comments have been addressed. However, in the spirit of using active voice, please revise line #82 to read "Therefore, the present study examined..." Whereas the current statement is (unfortunately) prevalent in the literature "Therefore, the purpose of the present study was to examine...", this is poor technical writing with the first 9 words not saying anything.

Reviewer #3: The authors have addressed all concerns by this reviewer. The authors should be commended for their updates to this manuscript.

**********

7. PLOS authors have the option to publish the peer review history of their article (what does this mean?). If published, this will include your full peer review and any attached files.

If you choose “no”, your identity will remain anonymous but your review may still be made public.

**Do you want your identity to be public for this peer review?**  For information about this choice, including consent withdrawal, please see our Privacy Policy.

Reviewer #1: No

Reviewer #2: No

Reviewer #3: No

**********

# Editor report: Acceptance letter

**DOI:** 10.1371/journal.pone.0352203.r004

**Authors:** William Adams

PONE-D-26-07092R1

PLOS One

Dear Dr. McDermott,

I'm pleased to inform you that your manuscript has been deemed suitable for publication in PLOS One. Congratulations! Your manuscript is now being handed over to our production team.

At this stage, our production department will prepare your paper for publication. This includes ensuring the following:

* All references, tables, and figures are properly cited

* All relevant supporting information is included in the manuscript submission,

* There are no issues that prevent the paper from being properly typeset

You will receive further instructions from the production team, including instructions on how to review your proof when it is ready. Please keep in mind that we are working through a large volume of accepted articles, so please give us a few days to review your paper and let you know the next and final steps.

Lastly, if your institution or institutions have a press office, please let them know about your upcoming paper now to help maximize its impact. If they'll be preparing press materials, please inform our press team within the next 48 hours. Your manuscript will remain under strict press embargo until 2 pm Eastern Time on the date of publication. For more information, please contact onepress@plos.org.

You will receive an invoice from PLOS for your publication fee after your manuscript has reached the completed accept phase. If you receive an email requesting payment before acceptance or for any other service, this may be a phishing scheme. Learn how to identify phishing emails and protect your accounts at https://explore.plos.org/phishing.

If we can help with anything else, please email us at customercare@plos.org.

Thank you for submitting your work to PLOS ONE and supporting open access.

Kind regards,

PLOS ONE Editorial Office Staff

on behalf of

Dr. William M. Adams

Academic Editor

PLOS One
